# Finasteride-Its Impact on Sexual Function and Prostate Cancer

**DOI:** 10.4103/0974-2077.53093

**Published:** 2009

**Authors:** B Anitha, Arun C Inamadar, S Ragunatha

**Affiliations:** *Department of Dermatology, Venereology and Leprosy, Shri B.M. Patil Medical College Hospital and Research Centre, Bijapur, Karnataka, India*

**Keywords:** Finasteride, prostate cancer, sexual function

## Abstract

Finasteride, a specific and competitive inhibitor of 5α-reductase enzyme Type 2, inhibits the conversion of testosterone to dihydrotestosterone (DHT). In adults, DHT acts as primary androgen in prostate and hair follicles. The only FDA-approved dermatological indication of finasteride is androgenetic alopecia. But, apprehension regarding sexual dysfunction associated with finasteride deters dermatologists from prescribing the drug and patients from taking the drug for androgenetic alopecia. Testosterone, through its humoral endocrine and local paracrine effects is relevant in central and peripheral modulation of sexual function than locally acting DHT. Several large population-based long-term placebo-controlled studies, using International Index of Erectile Function-5 questionnaire and objective method (Nocturnal Penile Tumescence) to assess the erectile function have demonstrated no clear evidence of the negative effect of finasteride on erectile function. Reduction in ejaculatory volume is the only established causal relationship between finasteride and sexual dysfunction. Though finasteride causes significant reduction in all the semen parameters except sperm morphology, they did not fall below the threshold levels to interfere with fertility. Therefore, the sexual adverse effects associated with finasteride should be viewed in relation to normal prevalence and natural history of erectile dysfunction in the population, age of the patient, other confounding factors and also nocebo effect. The impact of finasteride on the prevention of prostate cancer has been discussed extensively. Finasteride is found to be effective in significantly reducing the incidence of low-grade prostate cancer. But the paradoxical increase in high-grade cancer in the finasteride group has been attributed to increased sensitivity and improved performance of prostate specific antigen levels to detect all grades of prostate cancer.

## INTRODUCTION

Androgens, otherwise known as sex hormones, are essential for the development of external genitalia, testes, and maintenance of spermatogenesis and secondary sexual characters. Testosterone and dihydrotestosterone (DHT) are the main biologically active forms of androgens. Testosterone is synthesized by both gonads and adrenal glands. In the testes, testosterone is synthesized by the Leydig cells in response to stimulation by lutenizing hormone.[1] In men, 4-8% of testosterone is converted to the more potent androgen, DHT by the action of 5α-reductase enzyme.[2] During embryogenesis, testosterone plays a role in Wolffian ductal differentiation whereas DHT mediates male external genitalia and prostate differentiation.[3] In adults, DHT acts as primary androgen in prostate and hair follicles which tend to accelerate benign prostatic hypertrophy (BPH) and androgenetic alopecia.[2]

## PHARMACOLOGY OF FINASTERIDE

Finasteride is a specific and competitive inhibitor of Type 2 5α-reductase. The enzyme, 5α-reductase is required for conversion of testosterone to DHT. It exists in two isoenzyme forms. Type 1 is predominant in the sebaceous glands and liver, and Type 2 is predominant in the prostate, seminal vesicles, epididymes, hair follicles and liver.[3] Finasteride has no affinity for androgen receptor and hence has no androgen-related actions like androgenic, antiandrogenic, estrogenic, antiestrogenic or progestational effects.[4]

The administration of finasteride 5 mg/day for the treatment of BPH results in 60-93% reduction in circulating DHT levels from the baseline[1] with a 15 - 25% rise in testosterone levels.[2,5]. The effects of finasteride 5 mg and 1 mg/day result in almost similar changes in DHT and testosterone levels in serum, prostate and scalp skin. However, a significantly greater fall of prostate DHT levels after six to eight weeks of therapy has been reported in patients taking finasteride 5 mg/day compared to those taking finasteride 1 mg/day.[6]

The bioavailability of finasteride 1mg following oral intake ranges from 26-170% with a mean of 65%. The average peak plasma concentration has been found to be 9.2 ng/mL measured 1-2 h post dose. The bioavailability of finasteride is not related to food intake. Finasteride is extensively metabolized in the liver by Cytochrome P450 3A4 enzyme subfamily and excreted both in urine and feces. The terminal half-life is approximately 5-6 h in men between 18-60 years of age and 8 h in men more than 70 years of age.[4]

The FDA-approved dermatologic indication is male pattern androgenetic alopecia. Other dermatologic uses include hirsutism, acne vulgaris, and hidradenitis suppurativa. Various adverse effects of finasteride include sexual dysfunctions; hypersensitivity reactions such as rash, pruritus, urticaria, and swelling of the lips and face; breast tenderness and enlargement; severe myopathy and testicular pain. It is also known to have teratogenic effects in animals.[7]

## FINASTERIDE AND SEXUAL FUNCTION

To understand the impact of finasteride on sexual function, it is important to know the normal physiology of male sexual function and the role of testosterone and DHT in erectile function.

### Normal male sexual function

Male sexual function normally requires intact libido; the ability to achieve and maintain penile erection; ejaculation; and detumescence. Androgens, especially testosterone increases the libido. A variety of visual, olfactory, tactile, auditory and imaginative stimuli can also influence the libido. The penile erection is mainly under the control of the parasympathetic nervous system. The nitric oxide released from the non-adrenergic, non-cholinergic autonomic fibers causes relaxation of smooth muscles in the penis, leading to increased flow and accumulation of blood in the lacunar network of corpora which are converted into non-compressible cylinders resulting in erection. The nitric oxide is synthesized in the cavernosal tissue of penis by nitric oxide synthetase. Ejaculation and detumescence require intact sympathetic system.[8]

### Role of androgens in erectile function

The integrity of structural and cellular components of the penis, and veno-occlusive mechanism is essential for normal erectile function. It has been demonstrated that deprivation of testosterone results in apoptosis of cells from the cavernosal and spongiosal tissue. In animal experiments on castrated rats, the importance of testosterone in normalizing erectile function and nitric oxide synthetase activity has been demonstrated.[1] Even an individual with low testosterone levels can achieve erection. However, in elderly males, normal testosterone levels appear to be important for erection.[8] Unlike testosterone, DHT does not seem to affect the erectile function. DHT is a paracrine hormone exerting its action in the tissue of origin. The intact erectile function in the presence of low DHT levels in men with 5α- reductase enzyme deficiency and in men receiving finasteride, and restoration of erectile function in hypogonadal men in response to 7-alpha- methyl- 9-nortestosterone, a 5α-reductase-resistant androgen, suggests that conversion of testosterone to DHT is not necessary for penile erection.[1]

Thus, the testosterone, through its humoral endocrine and local paracrine effects is relevant in central and peripheral modulation of sexual function than locally acting DHT.[1]

### Finasteride and sexual dysfunction

A comprehensive literature review of all the publications concerning 5α-reductase inhibitors and sexual adverse effects has revealed that sexual adverse effects occur at the rates of 2.1-38%, erectile dysfunction (ED) being the commonest followed by ejaculatory dysfunction and loss of libido.[9]

### Erectile dysfunction

Review of the literature on ED in men taking finasteride revealed the incidence of ED to be between 0.8-33%. However, randomized controlled studies reported ED to be between 0.8-15.8%.[1]

The clinical studies which reported increased incidence of ED in patients taking finasteride (5 mg or 1 mg/ day) did not either assess the baseline sexual function or use a validated questionnaire.[10–12] The ED occurred predominantly during the first year of therapy and subsequently by the end of three to seven years of therapy it resolved completely in half of the patients.[13–15] Thus the prevalence of ED declined with increased duration of therapy. Therefore, large population-based long-term placebo-controlled clinical studies using a validated questionnaire and objective method of assessment of sexual function are required to establish the causal relationship between finasteride and ED.

The Proscar (Finasteride 5 mg/day) Long-term Efficacy and Safety Study (PLESS) where more than 3000 men with benign prostatic hyperplasia (BPH) were assessed over a period of four years concluded that mild to moderate ED which resolved in about half of the patients after discontinuation of either finasteride or placebo, is consistent with the natural history of ED in the patient population (>50 years) and concurrent substantial placebo effect.[15] In a double-blind placebo-controlled study using nocturnal penile tumescence (NPT) as an objective method of assessing erectile function, finasteride (5 mg/day for 12 weeks) failed to suppress consistent sleep- related penile erections.[16]

The clinical trials using International Index of Erectile Function-5 (IIEF-5) questionnaire to assess the sexual adverse effects of finasteride 1mg/day used in the treatment of male pattern hair loss have reported no statistically significant difference between the IIEF-5 scores obtained in patients receiving finasteride and placebo.[17,18] The ED due to finasteride has also been related to the nocebo effect i.e., an adverse effect that is not a direct result of the specific pharmacological action of the drug. In a study, the group informed about the sexual adverse effects of finasteride reported increased incidence of ED when compared to the group without such information.[19]

Dutasteride, a dual 5α-reductase enzyme inhibitor, reduces the DHT levels to a greater extent than finasteride. Several placebo-controlled studies have evaluated the efficacy and side-effects of dutasteride 0.5 mg/day in the treatment of BPH and prostate cancer. The ED has been reported to be significantly higher in the dutasteride group than in the placebo group (6.1% vs. 3.0%[20] and 4.7% vs. 1.7%[21]) during the first year of treatment. However, by the end of two years, the prevalence of ED was similar in both the groups (1.3% vs. 1.3%[20] and 0.8% vs. 0.9%[21]).

The analysis of results of the above mentioned clinical studies shows no clear evidence of negative effect of finasteride (5 mg or 1 mg/day) on erectile function. Therefore, ED occurring during finasteride therapy should be viewed in terms of normal prevalence and natural history of ED in the population, age of the patient, other confounding factors, and also the nocebo effect.

### Ejaculatory dysfunction

The effect of finasteride on ejaculatory volume and other semen parameters has not been reported in detail in the literature. The ejaculatory dysfunction associated with finasteride ranges from 2.1-7.7%.[22]

A randomized double-blind placebo-controlled study assessed the effect of finasteride (5 mg/day), dutasteride and placebo on the semen parameters of normal healthy men. The individuals received any one of these drugs for one year and semen analysis was performed at 26 weeks and 52 weeks of treatment, and also after 24 weeks of follow- up. The finasteride group showed statistically significant reduction in sperm count (34.3%), semen volume (21.1%) and sperm concentration (21.5%) from the baseline at 26 weeks of treatment. However, at 52 weeks of treatment and after 24 weeks follow-up, the reduction of all the three semen parameters from the baseline was no longer statistically significant. Finasteride also resulted in significant reduction in sperm motility of 6-12% from the baseline throughout the study period. But there was no significant change in sperm morphology anytime during the study.[2] The reduction in semen parameters in this study was slight and did not fall below the pre-established threshold levels to interfere with normal fertility. However, marked sensitivity of some individuals to finasteride may result in substantial reduction in semen quality leading to infertility. Therefore, finasteride should be considered as a possible etiological agent while evaluating men for infertility.[2]

Recovery of semen parameters towards the baseline at 52 weeks of treatment and after 24 weeks of follow-up in the presence of significant decrease in DHT levels (72.7%) suggests that DHT does not play a major role in spermatogenesis and testosterone alone may be sufficient to maintain spermatogenesis in normal healthy men.[2]

However, through its effect on size of prostate and seminal vesicles, finasteride results in low semen or ejaculatory volume. Finasteride causes significant reduction in prostate size through inhibition of conversion of testosterone to DHT by 5α-reductase Type 2 enzyme which is expressed in both epithelial and stromal cells of prostate but more predominantly in the latter.[3] A 19-28% reduction in the prostate size from the baseline has been reported after six months therapy with finasteride 5 mg/day for BPH.[10,11,23–25] When used in the dose of 1 mg day for one year, finasteride resulted in 18% reduction in prostate size from the baseline.[10]

A similar situation has been observed in men with congenital deficiency of Type 2 5α-reductase enzyme. In these patients, the prostate and seminal vesicles were atrophic with resulting low semen volume.[26]

Thus the only causal relationship between finasteride and sexual dysfunction is low semen or ejaculatory volume.

## FINASTERIDE AND PROSTATE CANCER

Prostate cancer is one of the common causes of cancer deaths in men. Most cancers develop in the peripheral zone of the prostate, and cancers in this location are palpable during digital rectal examination. The management of prostate cancer should be focused on early detection and treatment. However, prevention may be a more effective approach.[27]

The prostate epithelial cells and the stromal cells express androgen receptors and depend on androgens for growth.[27] Effectiveness of finasteride in the treatment of BPH had led to a hypothesis that finasteride may have a role in the prevention of prostate cancer.[6]

Finasteride was used in the Prostate Cancer Prevention Trial (PCPT), a double-blind, randomized multicenter trial. PCPT compared the ability of finasteride 5 mg versus placebo in reducing the risk of prostate cancer. The study reported that finasteride prevents or delays the appearance of prostate cancer, thereby decreasing the overall incidence of prostate cancer. Significantly, it was also found that there was an increase in the incidence of high-grade prostate cancer with finasteride compared to placebo. This may be due to the finasteride induced alteration of intraprostatic androgen levels leading to morphologic changes in low-grade tumors. Selective inhibition of low-grade tumor by finasteride may also be another explanation for the increase in high grade cancer.[28] More importantly, it is the effect of finasteride on levels of PSA which is responsible for early detection of increased number of high-grade prostate cancer. Finasteride (5 mg) has been found to reduce the PSA level by 50% from the baseline at the end of six months of therapy for BPH. Therefore, for prostate cancer screening, a compensatory adjustment of PSA level (multiplication by factor 2) is recommended in men who are on finasteride 5 mg/ day for BPH.[29] Similar reduction in PSA level has also been demonstrated in men aged between 40-60 years receiving finasteride 1mg/ day for androgenetic alopecia.[30] Hence, compensatory adjustment of PSA level should also be applied for these patients during prostate cancer screening. Higher PSA levels are usually associated with benign conditions like BPH and prostatitis than prostate cancer. Treatment with finasteride (5 mg/day) causes greatest fall in PSA levels in patients with BPH than prostate cancer. Patients receiving finasteride, who show persistent higher levels of PSA, are more likely to have cancer than those who are not on finasteride. The analysis of the PCPT study demonstrated that finasteride significantly increased the sensitivity of PSA levels in the detection of all grades of prostate cancer when compared to placebo. Thus increased incidence of high-grade prostate cancer in the finasteride group has been attributed to improved performance of PSA screening in detection of prostate cancer. The patients are more likely to have prostate cancer if finasteride does not reduce PSA level by 50%.[31]

However, there is a concern over chronic use of finasteride and development of prostate cancer. Through its effect on hormonal (estrogens vs. androgens) balance and immune surveillance of tumor cells, finasteride increases the risk of prostate cancer. In many studies it has been shown that the prostatic hyperplasia and cancer develop frequently in the hormonal milieu of estrogen excess over androgens. This hormonal imbalance is normally seen in aging males. Finasteride increases the circulating levels of testosterone which is peripherally aromatized to estrogens. Thus the use of finasteride in older males further shifts the hormonal balance towards estrogen excess. The expression of aromatase is also up-regulated in prostatic hyperplasia and cancer.

The immune competent cells possess androgen receptors and androgens are known to affect the Th1/Th2 balance. Finasteride alters the immune surveillance of cancer in aging males and may predispose them to the risk of prostate cancer.[32]

The supplementation of DHT, a non-aromatizing androgen, restores the estrogen-androgen balance by decreasing the plasma levels of estradiol and testosterone. It has been reported that DHT has a favorable effect on sexual function and the cardiovascular system without any adverse effects on prostate.[33] Paradoxically, the use of DHT has been proposed as an alternative treatment in the prevention of prostate cancer.[32] However, before any further therapeutic intervention to prevent or treat prostate cancer a clear understanding of androgen- and/or non-androgen-dependent mechanisms in the development prostate cancer is necessary.

## CONCLUSION

Although a relationship has been established between finasteride and sexual dysfunction in the literature, the analysis of the role of androgens in male sexual function and the evidences from large population-based long-term placebo-controlled studies using validated questionnaire and objective method for assessing sexual function suggested no substantial evidence of ED in men receiving finasteride. Low ejaculatory volume is the only causal relationship between finasteride and sexual dysfunction. Finasteride has been an effective drug in preventing low-grade prostate cancer but its role in increased incidence of high-grade prostate cancer has been attributed to better performance of PSA screening in prostate cancer detection. The effects of both doses of finasteride (5 mg and 1 mg/day) on prostate and PSA levels are almost similar. So, as dermatologists, we should be aware of the potential risks and benefits while treating baldness in young men with long-term finasteride.

## References

[1] Canguven O, Burnett AL (2008). The effect of 5α- reductase inhibitors on erectile function. J Androl.

[2] Amory JK, Wang C, Swerdloff RS, Anawalt BD, Matsumoto AM, Bremmer WJ (2007). The effect of 5α- reductase inhibition with dutasteride and finasteride on semen parameters and serum hormones in healthy men. J Clin Endocrinol Metab.

[3] Zhu YS, Sun GS (2005). 5α- reductase isozymes in the prostate. J Med Sci.

[4] Sudduth SL, Koronkowski MJ (1993). Finasteride: The first 5α- reductase inhibitor. Pharmacotherapy.

[5] Uygur MC, Arik AI, Altug U, Erol D (1998). Effects of the 5α- reductase inhibitor finasteride on serum levels of gonadal, adrenal, and hypophyseal hormones and its clinical significance: A prospective clinical study. Steroids.

[6] Rosner W (2004). Proscar and Propecia - A therapeutic perspective. J Clin Endocrinol Metab.

[7] Sawaya ME, Wolverton SE (2007). Antiandrogens and androgen inhibitors. Comprehensive Dermatologic drug therapy.

[8] McVary KT, Fauci AS, Braunwald E, Kasper DL, Hauser SL, Longo DL, Jameson JL, Loscalzo J (2008). Sexual dysfunction. Harrison's Principles of Internal Medicine.

[9] Erdemir F, Harbin A, Hellstorm WJ (2008). 5α reductase inhibitors and erectile dysfunction: The connection. J Sex Med.

[10] Gormley GJ, Stoner E, Bruskewitz RC, Imperato-McGinley J, Walsh PC, McConnell JD (1992). The effect of finasteride in men with benign prostatic hyperplasia: The Finasteride study group. N Engl J Med.

[11] Nickel JC, Fradet Y, Boake RC, Pommerville PJ, Perreault JP, Afridi SK (1996). Efficacy and safety of finasteride therapy for benign prostatic hyperplasia: Results of a 2-year randomized controlled trial (the PROSPECT study). Proscar Safety Plus Efficacy Canadian Two year study. CMAJ.

[12] Debruyne FM, Jardin A, Colloi D, Resel L, Witjes WP, Delauche- Cavallier MC (1998). Sustained- release alfuzosin, finasteride and combination of both in the treatment of benign prostatic hyperplasia. European ALFIN study group. Eur Urol.

[13] Stoner E (1994). Three- year safety and efficacy data on the use of finasteride in the treatment of benign prostatic hyperplasia. Urology.

[14] Moinpour CM, Darke AK, Donaldson GW, Thompson IM, Langley C, Ankerst DP (2007). Longitudinal analysis of sexual function reported by men in the prostate cancer prevention trial. J Natl Cancer Inst.

[15] Wessels H, Roy J, Bannow J, Grayhack J, Matsumoto AM, Tenover L (2003). Incidence and severity of sexual adverse experiences in finasteride and placebo treated men with benign prostatic hyperplasia. Urology.

[16] Cunningham GR, Hirshkowitz M (1995). Inhibition of steroid 5 alpha- reductase with finasteride: Sleep- related erections, potency, and libido in healthy men. J Clin Endocrinol Metab.

[17] Tosti A, Pazzaglia M, Soli M, Rossi A, Rebora A, Atzori L (2004). Evaluation of sexual function with an International Index of Erectile Function in subjects taking finasteride for androgenetic alopecia. Arch Dermatol.

[18] Tosti A, Pirracini BM, Soli M (2001). Evaluation of sexual function in subjects taking finasteride for androgenetic alopecia. J Eur Acad Dermatol Venereol.

[19] Mondaini N, Gontero P, Giubilei G, Lombardi G, Cai T, Gavazzi A (2007). Finasteride 5mg and sexual side effects: How many of these are related to a nocebo phenomenon?. J Sex Med.

[20] Roehrborn CG, Marks LS, Fenter T, Freedman S, Tuttle J, Gittleman M (2004). Efficacy and safety of dutasteride in the four- year treatment of men with benign prostatic hyperplasia. Urology.

[21] Andriole GL, Kirby R (2003). Safety and tolerability of the dual 5 alpha- reductase inhibitor dutasteride in the treatment of benign prostatic hyperplasia. Eur Urol.

[22] Carbone DJ, Hodges S (2003). Medical therapy for benign prostatic hyperplasia. Sexual dysfunction and impact on quality of life. International Journal of Impotence Research.

[23] Stoner E (1990). The clinical development of a 5α- reductase inhibitor. Finasteride. J Steroid Biochem Mol Biol.

[24] Ekman P (1998). A risk- benefit assessment of treatment with finasteride in benign prostatic hyperplasia. Drug Saf.

[25] Ekman P (1999). Finasteride in the treatment of benign prostatic hyperplasia: An update. New indication for finasteride therapy. Scand J Urol Nephrol Suppl.

[26] Cai LQ, Fratianni CM, Gautier T, Imperato- McGinley J (1994). Dihydrotestosterone regulation of semen in male pseudohermaphrodites with 5α- reductase- 2 deficiency. J Clin Endocrinol Metab.

[27] Scher HI, Fauci AS, Braunwald E, Kasper DL, Hauser SL, Longo DL, Jameson JL, Loscalzo J (2008). Benign and malignant diseases of the prostate. Harrison's Principles of Internal Medicine.

[28] Thompson MI, Goodman PJ, Tangen CM, Lucia MS, Miller GJ, Ford LG (2003). Influence of finasteride on the development of prostate cancer. N Engl J Med.

[29] Hendel LN, Agarwal S, Schiff SF, Kelty PJ, Cohen SI (2006). Can effect of finasteride on prostate specific antigen be used to decrease repeat prostate biopsy?. Urology.

[30] D'Amico AV, Roehrborn CG (2007). Effect of 1mg/ day finasteride on concentration of serum prostate specific antigen in men with androgenetic alopecia: A randomized controlled trial. Lancet Oncol.

[31] Thompson IM, Chi C, Ankerst DP, Goodman PJ, Tangen CM, Lippman SM (2006). Effect of finasteride on the sensitivity of PSA for detecting prostate cancer. J Natl Cancer Inst.

[32] Palusinski R, Barud W (2004). Proscar and Propecia - A therapeutic perspective. Clin Endocrinol Metab.

[33] Barud W, Palusinski R, Makaruk B, Hanzlik J (2003). Dihydrotestosterone treatment in men with coronary artery disease. Influence on sex hormones, lipid profile, insulin resistance and fibrynogen. Annales UMCS Sectio D.

